# Di-μ-bromido-tris­(triphenyl­phosphine)-1κ*P*,2κ^2^ *P*-disilver(I) tetra­hydro­furan 0.85-solvate

**DOI:** 10.1107/S1600536809010241

**Published:** 2009-03-25

**Authors:** Weiping Gao, Yanhong Zhou, Hong Zhang

**Affiliations:** a Jilin Institute of Chemical Technology, Jilin 132000, People’s Republic of China; b Faculty of Chemistry, Northeast Normal University, Changchun 130024, People’s Republic of China

## Abstract

In the title binuclear silver(I) complex, [Ag_2_Br_2_(C_18_H_15_P)_3_]·0.85C_4_H_8_O, the two independent silver(I) ions are briged by two bromide ions. One Ag^I^ ion is coordinated by two triphenyl­phosphine groups with a square-planar geometry, while the second is coordinated by one triphenyl­phine group with a trigonal-planar geometry. The structure is very similar to that of the dichloro­methane solvate reported by Zhu, Huang & Zheng [*Chin. J. Struct. Chem.* (1994), **13**, 325–327]. The tetrahydrofuran solvent molecule is disordered and was refined with a fixed occupancy of 0.85.

## Related literature

For the structure of the dichloro­methane solvate, see: Zhu *et al.* (1994 ▶). For general background on triphenyl­phosphine–silver(I) complexes, see: Whitcomb & Rajeswaram (2006 ▶); Whitcomb & Roger (1996 ▶); Mann *et al.* (1937 ▶); Teo & Calabrese (1976 ▶); Bowmaker *et al.* (1993 ▶); Olmstead *et al.* (2004 ▶); Zhang *et al.* (2003 ▶); Lobana *et al.* (2008 ▶); Cingolani *et al.* (2003 ▶). For the sensitization of photothermographic materials by coordination polymers formed by the reaction of triphenyl­phosphine with silver(I) salts, see: Freedman (1994 ▶).
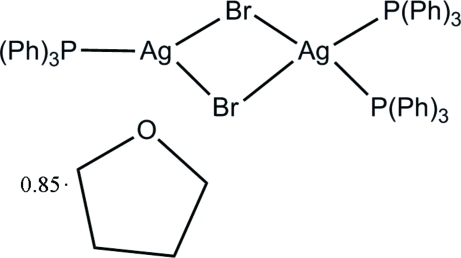


## Experimental

### 

#### Crystal data

[Ag_2_Br_2_(C_18_H_15_P)_3_]·0.85C_4_H_8_O
*M* *_r_* = 1223.66
Monoclinic, 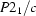

*a* = 16.2386 (19) Å
*b* = 19.4575 (17) Å
*c* = 18.3069 (18) Å
β = 111.896 (1)°
*V* = 5367.0 (9) Å^3^
*Z* = 4
Mo *K*α radiation
μ = 2.35 mm^−1^
*T* = 293 K
0.40 × 0.12 × 0.08 mm

#### Data collection

Rigaku Mercury CCD diffractometer
Absorption correction: multi-scan (SPHERE in *CrystalClear*; Rigaku, 2002 ▶) *T*_min_ = 0.721, *T*_max_ = 0.829
36137 measured reflections
9804 independent reflections
4750 reflections with *I* > 2σ(*I*)
*R*_int_ = 0.095

#### Refinement

*R*[*F* ^2^ > 2σ(*F* ^2^)] = 0.076
*wR*(*F* ^2^) = 0.115
*S* = 1.00
9804 reflections
596 parameters
H-atom parameters constrained
Δρ_max_ = 0.37 e Å^−3^
Δρ_min_ = −0.41 e Å^−3^

### 

Data collection: *CrystalClear* (Rigaku, 2002 ▶); cell refinement: *CrystalClear*; data reduction: *CrystalClear*; program(s) used to solve structure: *SHELXS97* (Sheldrick, 2008 ▶); program(s) used to refine structure: *SHELXL97* (Sheldrick, 2008 ▶); molecular graphics: *SHELXTL* (Sheldrick, 2008 ▶); software used to prepare material for publication: *SHELXTL*.

## Supplementary Material

Crystal structure: contains datablocks I, global. DOI: 10.1107/S1600536809010241/su2091sup1.cif

Structure factors: contains datablocks I. DOI: 10.1107/S1600536809010241/su2091Isup2.hkl

Additional supplementary materials:  crystallographic information; 3D view; checkCIF report

## Figures and Tables

**Fig. 1.**
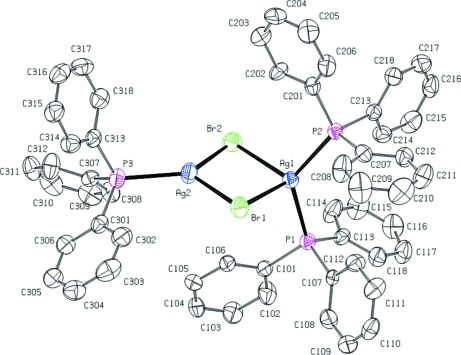
The molecular structure of compound (I) showing the thermal ellipsoids drawn at the 30% probabilty level (the H-atoms and the THF molecule of crystallization have been omitted for clarity).

## supplementary crystallographic information

### Comment

As triphenylphosphine is an electrically neutral molecule, it can react with silver (I) salts to form coordination polymers, some of which can prompt the sensitization of photothermographic (PTG) materials (Freedman, 1994). Since the first tertiary phosphine silver (I) complexes of the type [AgXL_n_] (L is tertiary phosphine; n is 1-4; X is a coordinating or non-coordinating anion) were prepared in 1937 by Mann *et al.* (1937), many complexes have been obtained by the reaction of triphenylphosphine with an appropriate silver (I) salt (Whitcomb & Rajeswaram, 2006; Whitcomb & Roger, 1996; Teo *et al.*, 1976; Bowmaker *et al.*, 1993; Olmstead *et al.*, 2004; Zhang *et al.*, 2003; Lobana *et al.*, 2008; Cingolani *et al.*, 2003). In this paper, we report on the tetrahydrofuran (THF) solvate of the binuclear triphenylphosphine silver(I) complex, (I), synthesized using AgBr.

The molecular structure of (I) is shown in Fig. 1. The structure is a solvo-polymorph of the dichloromethane analogue, (II), described by Zhu *et al.* (1994). The bond distances and angles in the complex are similar to those reported for compound (II). Complex (I) contains a rhomboid cyclic [Ag_2_Br_2_] moiety, with three coordinated triphenylphosphine molecules and a THF solvent molecule. One of the two silver(I) atoms, Ag1, is four-coordinated by two bridging bromine atoms and two P-atoms from two triphenylphosphines ligands, forming a distorted tetrahedral geometry. The other silver(I) atom, Ag2, is three-coordinated by two bridging bromine atoms and one P-atom from a triphenylphosphine ligand, to form an irregular trigonal plane.

### Experimental

In a glove box, AgBr (0.15 mmol, 0.028 g) and PPh_3_ (0.45 mmol, 0.118 g) were dissolved in 10 mL of distilled THF. The mixture was stirred for 2h to get a clear solution, then a little (3 ml) 1,2-bis(trimethylsilyl)ethyne was added to the above solution, and stirring was continued for three hours. The solution was then concentrated and the recipient sealed and placed in the refrigerator. After a few days colorless prismatic crystals of (I) were obtained.

### Refinement

The H-atoms were included in calculated positions and treated as riding atoms: C-H = 0.93 - 0.97 Å with U_iso_(H) = 1.2U_eq_(parent C-atom). The intensity data were measured at room temperature and the crystal did not diffract very strongly; less than 50% of the data can be considered to be observed [I>2σ(I)]. Hence, the bond distances and angles are not very precise and some C-atoms suffer from thermal disorder. A disordered THF molecule is present in the asymmetric unit: occupancy fixed at 0.85.

### Crystal data

**Table:**

[Ag_2_Br_2_(C_18_H_15_P)_3_]·0.85C_4_H8O	*F*(000) = 2448
*M**_r_* = 1223.66	*D*_x_ = 1.514 Mg m^−^^3^
Monoclinic, *P*2_1_/*c*	Mo *K*α radiation, λ = 0.71073 Å
Hall symbol: -P 2ybc	Cell parameters from 6309 reflections
*a* = 16.2386 (19) Å	θ = 3.1–27.5°
*b* = 19.4575 (17) Å	µ = 2.35 mm^−^^1^
*c* = 18.3069 (18) Å	*T* = 293 K
β = 111.896 (1)°	Prism, colorless
*V* = 5367.0 (9) Å^3^	0.40 × 0.12 × 0.08 mm
*Z* = 4	

### Data collection

**Table:**

Rigaku Mercury CCD diffractometer	9804 independent reflections
Radiation source: rotating-anode generator	4750 reflections with *I* > 2σ(*I*)
graphite	*R*_int_ = 0.095
ω scans	θ_max_ = 25.4°, θ_min_ = 3.1°
Absorption correction: multi-scan (SPHERE in *CrystalClear*; Rigaku, 2002)	*h* = −18→19
*T*_min_ = 0.721, *T*_max_ = 0.829	*k* = −23→22
36137 measured reflections	*l* = −22→21

### Refinement

**Table:**

Refinement on *F*^2^	Primary atom site location: structure-invariant direct methods
Least-squares matrix: full	Secondary atom site location: difference Fourier map
*R*[*F*^2^ > 2σ(*F*^2^)] = 0.076	Hydrogen site location: inferred from neighbouring sites
*wR*(*F*^2^) = 0.115	H-atom parameters constrained
*S* = 1.00	*w* = 1/[σ^2^(*F*_o_^2^) + (0.016*P*)^2^] where *P* = (*F*_o_^2^ + 2*F*_c_^2^)/3
9804 reflections	(Δ/σ)_max_ = 0.018
596 parameters	Δρ_max_ = 0.37 e Å^−^^3^
0 restraints	Δρ_min_ = −0.41 e Å^−^^3^

### Special details

**Table:**

Geometry. All esds (except the esd in the dihedral angle between two l.s. planes) are estimated using the full covariance matrix. The cell esds are taken into account individually in the estimation of esds in distances, angles and torsion angles; correlations between esds in cell parameters are only used when they are defined by crystal symmetry. An approximate (isotropic) treatment of cell esds is used for estimating esds involving l.s. planes.

### Fractional atomic coordinates and isotropic or equivalent isotropic displacement parameters (Å^2^)

**Table:**

	*x*	*y*	*z*	*U*_iso_*/*U*_eq_	Occ. (<1)
Ag1	0.172104 (16)	0.430186 (13)	0.311968 (13)	0.05624 (9)	
Ag2	0.309939 (18)	0.449839 (16)	0.211990 (14)	0.08013 (12)	
Br1	0.23309 (2)	0.549632 (18)	0.258758 (19)	0.06869 (13)	
Br2	0.26760 (2)	0.335663 (19)	0.264353 (19)	0.07471 (14)	
P1	0.01808 (6)	0.41825 (4)	0.21950 (4)	0.0519 (3)	
P2	0.22977 (5)	0.44191 (4)	0.45510 (4)	0.0485 (3)	
P3	0.38713 (6)	0.46569 (5)	0.12429 (5)	0.0626 (3)	
C101	0.00382 (19)	0.40749 (14)	0.11612 (15)	0.0508 (11)	
C102	−0.0673 (2)	0.37370 (17)	0.06109 (16)	0.0826 (15)	
H10A	−0.1113	0.3545	0.0758	0.099*	
C103	−0.0727 (2)	0.36858 (18)	−0.01647 (17)	0.0834 (15)	
H10B	−0.1214	0.3472	−0.0540	0.100*	
C104	−0.0071 (2)	0.39468 (16)	−0.03775 (16)	0.0739 (14)	
H10G	−0.0113	0.3909	−0.0896	0.089*	
C105	0.0644 (2)	0.42627 (16)	0.01636 (16)	0.0736 (13)	
H10F	0.1095	0.4434	0.0018	0.088*	
C106	0.06945 (18)	0.43261 (15)	0.09268 (14)	0.0571 (12)	
H10C	0.1184	0.4544	0.1294	0.068*	
C107	−0.04536 (17)	0.49553 (14)	0.22217 (14)	0.0456 (10)	
C108	−0.1150 (2)	0.52007 (16)	0.15705 (16)	0.0712 (13)	
H10D	−0.1318	0.4967	0.1094	0.085*	
C109	−0.1594 (2)	0.57895 (17)	0.16262 (17)	0.0831 (15)	
H10E	−0.2060	0.5953	0.1188	0.100*	
C110	−0.1347 (2)	0.61364 (16)	0.23307 (17)	0.0751 (13)	
H11A	−0.1646	0.6535	0.2367	0.090*	
C111	−0.0673 (2)	0.58987 (16)	0.29710 (18)	0.0751 (14)	
H11B	−0.0515	0.6129	0.3449	0.090*	
C112	−0.0221 (2)	0.53159 (15)	0.29149 (15)	0.0649 (13)	
H11C	0.0252	0.5163	0.3355	0.078*	
C113	−0.04710 (18)	0.34765 (15)	0.23595 (15)	0.0516 (11)	
C114	−0.0173 (2)	0.28130 (16)	0.23901 (18)	0.0875 (14)	
H11D	0.0355	0.2731	0.2318	0.105*	
C115	−0.0632 (2)	0.22693 (17)	0.25242 (19)	0.1044 (17)	
H11E	−0.0414	0.1825	0.2538	0.125*	
C116	−0.1392 (2)	0.23703 (16)	0.26362 (18)	0.0853 (15)	
H11F	−0.1704	0.2000	0.2725	0.102*	
C117	−0.1703 (2)	0.30293 (16)	0.26176 (17)	0.0840 (14)	
H11G	−0.2227	0.3108	0.2699	0.101*	
C118	−0.1237 (2)	0.35738 (15)	0.24780 (16)	0.0652 (12)	
H11H	−0.1455	0.4018	0.2465	0.078*	
C201	0.34954 (19)	0.44201 (15)	0.50661 (15)	0.0538 (11)	
C202	0.39821 (18)	0.40313 (15)	0.47389 (17)	0.0627 (13)	
H20E	0.3698	0.3802	0.4265	0.075*	
C203	0.4888 (2)	0.39844 (17)	0.51154 (18)	0.0853 (16)	
H20F	0.5208	0.3711	0.4898	0.102*	
C204	0.5329 (2)	0.43282 (19)	0.57997 (18)	0.0964 (17)	
H20G	0.5942	0.4295	0.6048	0.116*	
C205	0.4844 (2)	0.47214 (19)	0.61093 (19)	0.0981 (17)	
H20D	0.5135	0.4960	0.6575	0.118*	
C206	0.3944 (2)	0.47745 (18)	0.57535 (16)	0.0790 (15)	
H20A	0.3631	0.5051	0.5975	0.095*	
C207	0.1921 (2)	0.52024 (15)	0.48663 (15)	0.0567 (12)	
C208	0.2164 (2)	0.58233 (16)	0.46320 (19)	0.0945 (15)	
H20B	0.2534	0.5819	0.4347	0.113*	
C209	0.1880 (3)	0.6440 (2)	0.4805 (2)	0.1249 (19)	
H20C	0.2043	0.6847	0.4629	0.150*	
C210	0.1354 (3)	0.64572 (18)	0.5238 (2)	0.1132 (19)	
H21A	0.1167	0.6875	0.5371	0.136*	
C211	0.1107 (2)	0.58514 (17)	0.54747 (19)	0.0973 (15)	
H21B	0.0736	0.5859	0.5758	0.117*	
C212	0.1398 (2)	0.52266 (17)	0.53001 (16)	0.0704 (13)	
H21C	0.1235	0.4821	0.5480	0.084*	
C213	0.19096 (18)	0.37368 (14)	0.50264 (15)	0.0509 (11)	
C214	0.11738 (19)	0.33772 (15)	0.45777 (17)	0.0655 (13)	
H21D	0.0894	0.3487	0.4048	0.079*	
C215	0.0833 (2)	0.28525 (16)	0.48909 (18)	0.0841 (15)	
H21E	0.0315	0.2626	0.4584	0.101*	
C216	0.1273 (2)	0.26735 (16)	0.56627 (18)	0.0838 (14)	
H21F	0.1068	0.2314	0.5884	0.101*	
C217	0.2027 (2)	0.30337 (16)	0.61092 (17)	0.0752 (14)	
H21G	0.2324	0.2912	0.6634	0.090*	
C218	0.23466 (19)	0.35619 (15)	0.58029 (15)	0.0607 (12)	
H21H	0.2852	0.3800	0.6114	0.073*	
C301	0.3497 (2)	0.53937 (15)	0.05832 (16)	0.0585 (12)	
C302	0.3280 (2)	0.59806 (16)	0.08938 (19)	0.0826 (15)	
H30A	0.3289	0.5980	0.1405	0.099*	
C303	0.3048 (2)	0.65782 (18)	0.0436 (2)	0.0967 (17)	
H30B	0.2923	0.6980	0.0650	0.116*	
C304	0.3003 (2)	0.65737 (18)	−0.03249 (18)	0.0871 (16)	
H30C	0.2843	0.6970	−0.0630	0.105*	
C305	0.3194 (2)	0.59866 (17)	−0.06341 (18)	0.0798 (15)	
H30D	0.3155	0.5980	−0.1154	0.096*	
C306	0.3448 (2)	0.53976 (16)	−0.01771 (16)	0.0646 (13)	
H30E	0.3585	0.5001	−0.0392	0.077*	
C307	0.38065 (19)	0.39381 (15)	0.05913 (16)	0.0542 (12)	
C308	0.3072 (2)	0.35043 (15)	0.03950 (16)	0.0695 (13)	
H30F	0.2649	0.3570	0.0619	0.083*	
C309	0.2978 (2)	0.29778 (17)	−0.01327 (18)	0.0878 (16)	
H30G	0.2479	0.2698	−0.0273	0.105*	
C310	0.3590 (2)	0.28579 (15)	−0.04500 (18)	0.0881 (16)	
H31A	0.3517	0.2501	−0.0807	0.106*	
C311	0.4333 (2)	0.32783 (16)	−0.02323 (17)	0.0813 (14)	
H31B	0.4768	0.3195	−0.0439	0.098*	
C312	0.4440 (2)	0.38156 (16)	0.02830 (17)	0.0825 (14)	
H31C	0.4940	0.4094	0.0421	0.099*	
C313	0.50559 (19)	0.48006 (15)	0.17267 (15)	0.0552 (11)	
C314	0.5493 (2)	0.53755 (16)	0.16275 (16)	0.0694 (14)	
H31D	0.5175	0.5716	0.1280	0.083*	
C315	0.6389 (2)	0.54602 (18)	0.20288 (17)	0.0817 (15)	
H31E	0.6663	0.5856	0.1946	0.098*	
C316	0.6876 (2)	0.49874 (16)	0.25364 (17)	0.0793 (15)	
H31F	0.7481	0.5056	0.2810	0.095*	
C317	0.6473 (2)	0.44027 (17)	0.26461 (17)	0.0835 (15)	
H31G	0.6809	0.4060	0.2978	0.100*	
C318	0.5564 (2)	0.43179 (17)	0.22653 (17)	0.0737 (14)	
H31H	0.5291	0.3932	0.2372	0.088*	
O11	0.6007 (2)	0.26438 (16)	0.28899 (18)	0.1287 (16)	0.85
C11	0.5807 (3)	0.2330 (2)	0.2174 (3)	0.132 (2)	0.85
H11I	0.6179	0.1928	0.2228	0.158*	0.85
H11J	0.5908	0.2643	0.1804	0.158*	0.85
C12	0.4917 (3)	0.2138 (3)	0.1907 (3)	0.157 (3)	0.85
H12A	0.4571	0.2413	0.1454	0.188*	0.85
H12B	0.4851	0.1658	0.1754	0.188*	0.85
C13	0.4646 (3)	0.2242 (3)	0.2490 (3)	0.155 (3)	0.85
H13A	0.4518	0.1805	0.2681	0.186*	0.85
H13B	0.4106	0.2513	0.2309	0.186*	0.85
C14	0.5310 (3)	0.2589 (2)	0.3100 (2)	0.126 (2)	0.85
H14C	0.5104	0.3040	0.3177	0.151*	0.85
H14A	0.5468	0.2333	0.3588	0.151*	0.85

### Atomic displacement parameters (Å^2^)

**Table:**

	*U*^11^	*U*^22^	*U*^33^	*U*^12^	*U*^13^	*U*^23^
Ag1	0.04831 (14)	0.07159 (17)	0.04870 (12)	0.00071 (14)	0.01794 (10)	−0.00254 (13)
Ag2	0.05967 (17)	0.1225 (3)	0.06481 (15)	−0.00171 (18)	0.03082 (12)	−0.00557 (16)
Br1	0.0779 (2)	0.0623 (2)	0.07304 (19)	−0.0086 (2)	0.03638 (16)	−0.00307 (18)
Br2	0.0881 (3)	0.0738 (2)	0.0647 (2)	0.0226 (2)	0.03131 (17)	−0.00624 (19)
P1	0.0470 (5)	0.0608 (6)	0.0481 (4)	−0.0007 (5)	0.0180 (4)	−0.0051 (4)
P2	0.0496 (5)	0.0526 (5)	0.0450 (4)	−0.0035 (4)	0.0196 (3)	−0.0037 (4)
P3	0.0593 (5)	0.0730 (6)	0.0632 (5)	−0.0054 (5)	0.0318 (4)	−0.0106 (5)
C101	0.0480 (19)	0.057 (2)	0.0455 (16)	0.0037 (16)	0.0154 (14)	−0.0045 (15)
C102	0.084 (3)	0.109 (3)	0.0589 (19)	−0.021 (2)	0.0313 (17)	−0.0320 (19)
C103	0.074 (3)	0.112 (3)	0.058 (2)	−0.015 (2)	0.0182 (18)	−0.026 (2)
C104	0.085 (2)	0.095 (3)	0.0521 (17)	0.014 (2)	0.0371 (15)	−0.0034 (18)
C105	0.070 (2)	0.096 (3)	0.0567 (18)	0.025 (2)	0.0261 (15)	0.0230 (19)
C106	0.0438 (18)	0.079 (2)	0.0479 (16)	0.0015 (18)	0.0166 (14)	0.0018 (17)
C107	0.0373 (16)	0.060 (2)	0.0452 (15)	−0.0012 (15)	0.0218 (12)	0.0043 (15)
C108	0.072 (2)	0.083 (3)	0.0632 (19)	−0.004 (2)	0.0303 (16)	0.0070 (19)
C109	0.081 (2)	0.096 (3)	0.078 (2)	0.031 (2)	0.0355 (17)	0.022 (2)
C110	0.070 (2)	0.062 (2)	0.110 (2)	0.0080 (19)	0.0527 (16)	−0.0022 (19)
C111	0.065 (2)	0.068 (2)	0.088 (2)	−0.0039 (19)	0.0236 (18)	−0.0306 (19)
C112	0.060 (2)	0.075 (2)	0.0500 (18)	0.003 (2)	0.0098 (16)	−0.0131 (18)
C113	0.0396 (18)	0.061 (2)	0.0521 (17)	−0.0003 (16)	0.0147 (13)	−0.0077 (16)
C114	0.082 (2)	0.066 (2)	0.139 (2)	0.017 (2)	0.0688 (17)	0.005 (2)
C115	0.128 (3)	0.047 (2)	0.167 (3)	0.006 (2)	0.089 (2)	0.008 (2)
C116	0.099 (3)	0.061 (2)	0.104 (2)	−0.023 (2)	0.0473 (19)	−0.010 (2)
C117	0.083 (2)	0.079 (3)	0.111 (2)	−0.005 (2)	0.0613 (16)	0.010 (2)
C118	0.075 (2)	0.047 (2)	0.084 (2)	0.0006 (18)	0.0425 (15)	0.0128 (17)
C201	0.0556 (18)	0.054 (2)	0.0568 (16)	−0.0097 (16)	0.0271 (13)	0.0001 (16)
C202	0.0341 (19)	0.075 (2)	0.073 (2)	−0.0039 (17)	0.0140 (15)	−0.0096 (18)
C203	0.059 (2)	0.108 (3)	0.086 (2)	0.006 (2)	0.0237 (19)	0.007 (2)
C204	0.054 (3)	0.150 (4)	0.067 (2)	0.006 (3)	0.0009 (19)	0.023 (2)
C205	0.070 (3)	0.140 (3)	0.075 (2)	−0.037 (3)	0.018 (2)	−0.026 (2)
C206	0.061 (2)	0.121 (3)	0.0543 (19)	−0.024 (2)	0.0203 (16)	−0.026 (2)
C207	0.064 (2)	0.055 (2)	0.0489 (17)	−0.0067 (17)	0.0195 (15)	−0.0026 (16)
C208	0.120 (3)	0.062 (2)	0.131 (2)	−0.012 (2)	0.0817 (18)	−0.022 (2)
C209	0.175 (4)	0.076 (3)	0.154 (3)	−0.017 (3)	0.098 (2)	−0.021 (2)
C210	0.134 (3)	0.067 (3)	0.146 (3)	0.012 (2)	0.060 (2)	−0.033 (2)
C211	0.102 (3)	0.088 (3)	0.121 (2)	0.022 (2)	0.0646 (18)	−0.022 (2)
C212	0.079 (2)	0.069 (2)	0.0730 (19)	0.011 (2)	0.0386 (16)	0.0043 (18)
C213	0.0508 (18)	0.051 (2)	0.0545 (17)	0.0040 (16)	0.0242 (13)	0.0006 (15)
C214	0.055 (2)	0.070 (2)	0.0660 (19)	−0.0041 (19)	0.0163 (16)	0.0121 (18)
C215	0.071 (2)	0.056 (2)	0.130 (3)	−0.010 (2)	0.0440 (19)	0.009 (2)
C216	0.095 (2)	0.067 (2)	0.120 (2)	0.0102 (19)	0.0753 (16)	0.0272 (19)
C217	0.083 (2)	0.070 (2)	0.074 (2)	0.018 (2)	0.0315 (17)	0.0097 (19)
C218	0.054 (2)	0.062 (2)	0.0650 (19)	−0.0105 (18)	0.0212 (15)	0.0061 (17)
C301	0.0513 (19)	0.065 (2)	0.0597 (17)	−0.0014 (17)	0.0214 (14)	−0.0147 (17)
C302	0.074 (3)	0.091 (3)	0.080 (2)	0.009 (2)	0.0258 (18)	−0.005 (2)
C303	0.085 (3)	0.079 (3)	0.118 (3)	0.011 (2)	0.027 (2)	−0.033 (2)
C304	0.087 (3)	0.080 (3)	0.089 (2)	0.010 (2)	0.026 (2)	0.003 (2)
C305	0.066 (2)	0.094 (3)	0.079 (2)	−0.001 (2)	0.0267 (18)	0.005 (2)
C306	0.064 (2)	0.068 (2)	0.0592 (18)	−0.0032 (19)	0.0195 (16)	−0.0171 (17)
C307	0.0476 (19)	0.053 (2)	0.0595 (18)	−0.0010 (17)	0.0171 (15)	−0.0005 (16)
C308	0.079 (2)	0.065 (2)	0.0651 (19)	−0.012 (2)	0.0278 (17)	−0.0027 (18)
C309	0.097 (3)	0.074 (3)	0.080 (2)	−0.023 (2)	0.018 (2)	−0.003 (2)
C310	0.152 (3)	0.037 (2)	0.079 (2)	−0.016 (2)	0.047 (2)	−0.0093 (18)
C311	0.123 (3)	0.064 (2)	0.0759 (19)	0.002 (2)	0.0590 (17)	−0.0107 (18)
C312	0.095 (2)	0.077 (2)	0.095 (2)	−0.021 (2)	0.0578 (17)	−0.0362 (19)
C313	0.0551 (19)	0.064 (2)	0.0494 (16)	0.0007 (17)	0.0229 (14)	−0.0165 (16)
C314	0.061 (2)	0.069 (2)	0.074 (2)	−0.0044 (19)	0.0199 (17)	0.0019 (19)
C315	0.058 (2)	0.103 (3)	0.083 (2)	−0.013 (2)	0.0250 (18)	−0.007 (2)
C316	0.055 (2)	0.097 (3)	0.078 (2)	0.002 (2)	0.0154 (18)	−0.031 (2)
C317	0.081 (3)	0.084 (3)	0.071 (2)	0.028 (2)	0.0124 (19)	−0.003 (2)
C318	0.073 (2)	0.072 (2)	0.078 (2)	−0.001 (2)	0.0311 (17)	−0.011 (2)
O11	0.118 (3)	0.128 (3)	0.129 (2)	−0.019 (2)	0.033 (2)	0.021 (2)
C11	0.116 (3)	0.134 (5)	0.177 (4)	0.002 (3)	0.093 (3)	0.020 (3)
C12	0.130 (5)	0.173 (5)	0.150 (4)	0.014 (4)	0.031 (4)	−0.054 (4)
C13	0.072 (4)	0.142 (5)	0.247 (6)	0.009 (4)	0.056 (4)	0.011 (5)
C14	0.167 (5)	0.124 (4)	0.090 (3)	0.049 (4)	0.051 (3)	0.051 (3)

### Geometric parameters (Å, °)

**Table:**

Ag1—P2	2.4423 (8)	C209—C210	1.365 (6)
Ag1—P1	2.4530 (8)	C209—H20C	0.9300
Ag1—Br2	2.7487 (5)	C210—C211	1.367 (5)
Ag1—Br1	2.8374 (5)	C210—H21A	0.9300
Ag2—P3	2.3968 (11)	C211—C212	1.384 (5)
Ag2—Br2	2.6112 (5)	C211—H21B	0.9300
Ag2—Br1	2.6186 (5)	C212—H21C	0.9300
P1—C113	1.826 (3)	C213—C214	1.364 (4)
P1—C101	1.831 (3)	C213—C218	1.374 (3)
P1—C107	1.833 (3)	C214—C215	1.383 (5)
P2—C207	1.815 (3)	C214—H21D	0.9300
P2—C201	1.818 (3)	C215—C216	1.369 (4)
P2—C213	1.824 (3)	C215—H21E	0.9300
P3—C313	1.814 (3)	C216—C217	1.382 (4)
P3—C307	1.816 (3)	C216—H21F	0.9300
P3—C301	1.826 (3)	C217—C218	1.363 (4)
C101—C106	1.378 (4)	C217—H21G	0.9300
C101—C102	1.383 (4)	C218—H21H	0.9300
C102—C103	1.393 (4)	C301—C306	1.364 (4)
C102—H10A	0.9300	C301—C302	1.378 (4)
C103—C104	1.361 (5)	C302—C303	1.400 (5)
C103—H10B	0.9300	C302—H30A	0.9300
C104—C105	1.360 (4)	C303—C304	1.368 (5)
C104—H10G	0.9300	C303—H30B	0.9300
C105—C106	1.374 (4)	C304—C305	1.361 (5)
C105—H10F	0.9300	C304—H30C	0.9300
C106—H10C	0.9300	C305—C306	1.388 (4)
C107—C112	1.374 (4)	C305—H30D	0.9300
C107—C108	1.387 (3)	C306—H30E	0.9300
C108—C109	1.377 (5)	C307—C312	1.366 (5)
C108—H10D	0.9300	C307—C308	1.394 (4)
C109—C110	1.376 (4)	C308—C309	1.377 (4)
C109—H10E	0.9300	C308—H30F	0.9300
C110—C111	1.353 (4)	C309—C310	1.346 (6)
C110—H11A	0.9300	C309—H30G	0.9300
C111—C112	1.375 (4)	C310—C311	1.387 (5)
C111—H11B	0.9300	C310—H31A	0.9300
C112—H11C	0.9300	C311—C312	1.375 (4)
C113—C118	1.354 (5)	C311—H31B	0.9300
C113—C114	1.372 (4)	C312—H31C	0.9300
C114—C115	1.368 (5)	C313—C314	1.373 (4)
C114—H11D	0.9300	C313—C318	1.389 (4)
C115—C116	1.338 (5)	C314—C315	1.373 (4)
C115—H11E	0.9300	C314—H31D	0.9300
C116—C117	1.374 (4)	C315—C316	1.337 (4)
C116—H11F	0.9300	C315—H31E	0.9300
C117—C118	1.380 (5)	C316—C317	1.365 (5)
C117—H11G	0.9300	C316—H31F	0.9300
C118—H11H	0.9300	C317—C318	1.387 (4)
C201—C206	1.381 (4)	C317—H31G	0.9300
C201—C202	1.382 (4)	C318—H31H	0.9300
C202—C203	1.375 (4)	O11—C14	1.328 (6)
C202—H20E	0.9300	O11—C11	1.371 (5)
C203—C204	1.364 (4)	C11—C12	1.392 (7)
C203—H20F	0.9300	C11—H11I	0.9700
C204—C205	1.363 (5)	C11—H11J	0.9700
C204—H20G	0.9300	C12—C13	1.314 (8)
C205—C206	1.365 (5)	C12—H12A	0.9700
C205—H20D	0.9300	C12—H12B	0.9700
C206—H20A	0.9300	C13—C14	1.402 (6)
C207—C212	1.364 (5)	C13—H13A	0.9700
C207—C208	1.388 (4)	C13—H13B	0.9700
C208—C209	1.366 (5)	C14—H14C	0.9700
C208—H20B	0.9300	C14—H14A	0.9700
			
P2—Ag1—P1	129.31 (3)	C210—C209—H20C	120.2
P2—Ag1—Br2	111.52 (2)	C208—C209—H20C	120.2
P1—Ag1—Br2	105.57 (2)	C209—C210—C211	119.0 (4)
P2—Ag1—Br1	104.39 (2)	C209—C210—H21A	120.5
P1—Ag1—Br1	103.72 (2)	C211—C210—H21A	120.5
Br2—Ag1—Br1	97.398 (16)	C210—C211—C212	121.3 (4)
P3—Ag2—Br2	129.08 (3)	C210—C211—H21B	119.4
P3—Ag2—Br1	123.75 (3)	C212—C211—H21B	119.4
Br2—Ag2—Br1	106.740 (18)	C207—C212—C211	120.3 (3)
Ag2—Br1—Ag1	76.985 (14)	C207—C212—H21C	119.8
Ag2—Br2—Ag1	78.701 (15)	C211—C212—H21C	119.8
C113—P1—C101	103.10 (13)	C214—C213—C218	119.6 (3)
C113—P1—C107	104.27 (14)	C214—C213—P2	117.8 (2)
C101—P1—C107	105.40 (12)	C218—C213—P2	122.5 (2)
C113—P1—Ag1	117.50 (8)	C213—C214—C215	121.7 (3)
C101—P1—Ag1	115.31 (10)	C213—C214—H21D	119.2
C107—P1—Ag1	110.02 (8)	C215—C214—H21D	119.2
C207—P2—C201	104.85 (13)	C216—C215—C214	118.7 (3)
C207—P2—C213	104.01 (15)	C216—C215—H21E	120.7
C201—P2—C213	104.27 (13)	C214—C215—H21E	120.7
C207—P2—Ag1	112.33 (9)	C215—C216—C217	119.2 (3)
C201—P2—Ag1	117.70 (10)	C215—C216—H21F	120.4
C213—P2—Ag1	112.43 (9)	C217—C216—H21F	120.4
C313—P3—C307	103.33 (14)	C218—C217—C216	121.9 (3)
C313—P3—C301	103.47 (14)	C218—C217—H21G	119.1
C307—P3—C301	104.29 (13)	C216—C217—H21G	119.1
C313—P3—Ag2	114.64 (10)	C217—C218—C213	118.9 (3)
C307—P3—Ag2	115.10 (11)	C217—C218—H21H	120.6
C301—P3—Ag2	114.55 (12)	C213—C218—H21H	120.6
C106—C101—C102	118.3 (3)	C306—C301—C302	119.3 (3)
C106—C101—P1	117.83 (19)	C306—C301—P3	123.9 (2)
C102—C101—P1	123.8 (3)	C302—C301—P3	116.8 (2)
C101—C102—C103	119.6 (3)	C301—C302—C303	119.7 (3)
C101—C102—H10A	120.2	C301—C302—H30A	120.2
C103—C102—H10A	120.2	C303—C302—H30A	120.2
C104—C103—C102	120.5 (3)	C304—C303—C302	120.3 (3)
C104—C103—H10B	119.8	C304—C303—H30B	119.9
C102—C103—H10B	119.8	C302—C303—H30B	119.9
C105—C104—C103	120.5 (3)	C305—C304—C303	119.7 (3)
C105—C104—H10G	119.8	C305—C304—H30C	120.2
C103—C104—H10G	119.8	C303—C304—H30C	120.2
C104—C105—C106	119.5 (3)	C304—C305—C306	120.3 (3)
C104—C105—H10F	120.3	C304—C305—H30D	119.8
C106—C105—H10F	120.3	C306—C305—H30D	119.8
C105—C106—C101	121.7 (3)	C301—C306—C305	120.7 (3)
C105—C106—H10C	119.2	C301—C306—H30E	119.6
C101—C106—H10C	119.2	C305—C306—H30E	119.6
C112—C107—C108	118.3 (3)	C312—C307—C308	119.4 (3)
C112—C107—P1	118.66 (19)	C312—C307—P3	122.5 (2)
C108—C107—P1	123.1 (2)	C308—C307—P3	118.0 (3)
C109—C108—C107	120.3 (3)	C309—C308—C307	119.4 (3)
C109—C108—H10D	119.9	C309—C308—H30F	120.3
C107—C108—H10D	119.9	C307—C308—H30F	120.3
C110—C109—C108	120.0 (3)	C310—C309—C308	121.7 (3)
C110—C109—H10E	120.0	C310—C309—H30G	119.2
C108—C109—H10E	120.0	C308—C309—H30G	119.2
C111—C110—C109	120.2 (3)	C309—C310—C311	118.5 (3)
C111—C110—H11A	119.9	C309—C310—H31A	120.8
C109—C110—H11A	119.9	C311—C310—H31A	120.8
C110—C111—C112	120.0 (3)	C312—C311—C310	121.2 (4)
C110—C111—H11B	120.0	C312—C311—H31B	119.4
C112—C111—H11B	120.0	C310—C311—H31B	119.4
C107—C112—C111	121.3 (2)	C307—C312—C311	119.7 (3)
C107—C112—H11C	119.4	C307—C312—H31C	120.2
C111—C112—H11C	119.4	C311—C312—H31C	120.2
C118—C113—C114	117.1 (3)	C314—C313—C318	116.4 (3)
C118—C113—P1	123.0 (2)	C314—C313—P3	124.6 (2)
C114—C113—P1	119.8 (3)	C318—C313—P3	118.9 (2)
C115—C114—C113	121.8 (3)	C315—C314—C313	121.6 (3)
C115—C114—H11D	119.1	C315—C314—H31D	119.2
C113—C114—H11D	119.1	C313—C314—H31D	119.2
C116—C115—C114	120.6 (3)	C316—C315—C314	121.6 (3)
C116—C115—H11E	119.7	C316—C315—H31E	119.2
C114—C115—H11E	119.7	C314—C315—H31E	119.2
C115—C116—C117	119.0 (3)	C315—C316—C317	118.9 (3)
C115—C116—H11F	120.5	C315—C316—H31F	120.5
C117—C116—H11F	120.5	C317—C316—H31F	120.5
C116—C117—C118	120.0 (3)	C316—C317—C318	120.3 (3)
C116—C117—H11G	120.0	C316—C317—H31G	119.9
C118—C117—H11G	120.0	C318—C317—H31G	119.9
C113—C118—C117	121.5 (3)	C317—C318—C313	121.1 (3)
C113—C118—H11H	119.3	C317—C318—H31H	119.5
C117—C118—H11H	119.3	C313—C318—H31H	119.5
C206—C201—C202	118.4 (3)	C14—O11—C11	108.5 (3)
C206—C201—P2	124.9 (3)	O11—C11—C12	107.4 (4)
C202—C201—P2	116.67 (19)	O11—C11—H11I	110.2
C203—C202—C201	119.8 (3)	C12—C11—H11I	110.2
C203—C202—H20E	120.1	O11—C11—H11J	110.2
C201—C202—H20E	120.1	C12—C11—H11J	110.2
C204—C203—C202	121.6 (3)	H11I—C11—H11J	108.5
C204—C203—H20F	119.2	C13—C12—C11	107.0 (4)
C202—C203—H20F	119.2	C13—C12—H12A	110.3
C205—C204—C203	118.1 (3)	C11—C12—H12A	110.3
C205—C204—H20G	121.0	C13—C12—H12B	110.3
C203—C204—H20G	121.0	C11—C12—H12B	110.3
C204—C205—C206	121.7 (3)	H12A—C12—H12B	108.6
C204—C205—H20D	119.1	C12—C13—C14	109.2 (5)
C206—C205—H20D	119.1	C12—C13—H13A	109.8
C205—C206—C201	120.3 (3)	C14—C13—H13A	109.8
C205—C206—H20A	119.9	C12—C13—H13B	109.8
C201—C206—H20A	119.9	C14—C13—H13B	109.8
C212—C207—C208	117.5 (3)	H13A—C13—H13B	108.3
C212—C207—P2	124.9 (2)	O11—C14—C13	107.1 (4)
C208—C207—P2	117.7 (3)	O11—C14—H14C	110.3
C209—C208—C207	122.2 (4)	C13—C14—H14C	110.3
C209—C208—H20B	118.9	O11—C14—H14A	110.3
C207—C208—H20B	118.9	C13—C14—H14A	110.3
C210—C209—C208	119.7 (4)	H14C—C14—H14A	108.6
			
P3—Ag2—Br1—Ag1	169.76 (3)	P2—C201—C202—C203	−177.2 (3)
Br2—Ag2—Br1—Ag1	−3.346 (12)	C201—C202—C203—C204	−1.7 (5)
P2—Ag1—Br1—Ag2	117.60 (2)	C202—C203—C204—C205	0.4 (6)
P1—Ag1—Br1—Ag2	−105.01 (3)	C203—C204—C205—C206	0.1 (6)
Br2—Ag1—Br1—Ag2	3.069 (11)	C204—C205—C206—C201	0.7 (6)
P3—Ag2—Br2—Ag1	−169.18 (3)	C202—C201—C206—C205	−2.0 (5)
Br1—Ag2—Br2—Ag1	3.432 (12)	P2—C201—C206—C205	177.7 (3)
P2—Ag1—Br2—Ag2	−111.76 (3)	C201—P2—C207—C212	−114.6 (2)
P1—Ag1—Br2—Ag2	103.47 (3)	C213—P2—C207—C212	−5.4 (3)
Br1—Ag1—Br2—Ag2	−3.058 (11)	Ag1—P2—C207—C212	116.4 (2)
P2—Ag1—P1—C113	−55.84 (13)	C201—P2—C207—C208	67.2 (2)
Br2—Ag1—P1—C113	80.24 (12)	C213—P2—C207—C208	176.4 (2)
Br1—Ag1—P1—C113	−177.89 (12)	Ag1—P2—C207—C208	−61.8 (2)
P2—Ag1—P1—C101	−177.78 (10)	C212—C207—C208—C209	−1.6 (4)
Br2—Ag1—P1—C101	−41.69 (11)	P2—C207—C208—C209	176.7 (2)
Br1—Ag1—P1—C101	60.17 (11)	C207—C208—C209—C210	1.5 (5)
P2—Ag1—P1—C107	63.21 (11)	C208—C209—C210—C211	−1.5 (5)
Br2—Ag1—P1—C107	−160.71 (10)	C209—C210—C211—C212	1.7 (5)
Br1—Ag1—P1—C107	−58.84 (10)	C208—C207—C212—C211	1.7 (4)
P1—Ag1—P2—C207	−63.49 (13)	P2—C207—C212—C211	−176.5 (2)
Br2—Ag1—P2—C207	162.41 (12)	C210—C211—C212—C207	−1.8 (4)
Br1—Ag1—P2—C207	58.29 (12)	C207—P2—C213—C214	102.5 (3)
P1—Ag1—P2—C201	174.61 (11)	C201—P2—C213—C214	−147.8 (3)
Br2—Ag1—P2—C201	40.51 (11)	Ag1—P2—C213—C214	−19.2 (3)
Br1—Ag1—P2—C201	−63.61 (11)	C207—P2—C213—C218	−79.7 (3)
P1—Ag1—P2—C213	53.42 (12)	C201—P2—C213—C218	29.9 (3)
Br2—Ag1—P2—C213	−80.67 (11)	Ag1—P2—C213—C218	158.5 (2)
Br1—Ag1—P2—C213	175.20 (11)	C218—C213—C214—C215	2.3 (5)
Br2—Ag2—P3—C313	−95.32 (11)	P2—C213—C214—C215	−179.9 (3)
Br1—Ag2—P3—C313	93.20 (11)	C213—C214—C215—C216	−3.0 (5)
Br2—Ag2—P3—C307	24.36 (10)	C214—C215—C216—C217	1.8 (5)
Br1—Ag2—P3—C307	−147.13 (10)	C215—C216—C217—C218	−0.2 (5)
Br2—Ag2—P3—C301	145.26 (10)	C216—C217—C218—C213	−0.4 (5)
Br1—Ag2—P3—C301	−26.22 (11)	C214—C213—C218—C217	−0.6 (5)
C113—P1—C101—C106	−154.1 (2)	P2—C213—C218—C217	−178.3 (3)
C107—P1—C101—C106	96.8 (2)	C313—P3—C301—C306	91.1 (3)
Ag1—P1—C101—C106	−24.7 (3)	C307—P3—C301—C306	−16.7 (3)
C113—P1—C101—C102	23.6 (3)	Ag2—P3—C301—C306	−143.4 (2)
C107—P1—C101—C102	−85.4 (3)	C313—P3—C301—C302	−86.9 (3)
Ag1—P1—C101—C102	153.0 (2)	C307—P3—C301—C302	165.3 (3)
C106—C101—C102—C103	−2.7 (5)	Ag2—P3—C301—C302	38.6 (3)
P1—C101—C102—C103	179.5 (2)	C306—C301—C302—C303	−2.5 (5)
C101—C102—C103—C104	2.0 (5)	P3—C301—C302—C303	175.6 (3)
C102—C103—C104—C105	−0.2 (5)	C301—C302—C303—C304	2.4 (5)
C103—C104—C105—C106	−1.0 (5)	C302—C303—C304—C305	−0.6 (5)
C104—C105—C106—C101	0.2 (5)	C303—C304—C305—C306	−1.1 (5)
C102—C101—C106—C105	1.6 (4)	C302—C301—C306—C305	0.8 (5)
P1—C101—C106—C105	179.5 (2)	P3—C301—C306—C305	−177.1 (2)
C113—P1—C107—C112	96.1 (3)	C304—C305—C306—C301	1.0 (5)
C101—P1—C107—C112	−155.7 (3)	C313—P3—C307—C312	−28.7 (3)
Ag1—P1—C107—C112	−30.8 (3)	C301—P3—C307—C312	79.2 (3)
C113—P1—C107—C108	−85.0 (3)	Ag2—P3—C307—C312	−154.4 (2)
C101—P1—C107—C108	23.2 (3)	C313—P3—C307—C308	152.2 (2)
Ag1—P1—C107—C108	148.1 (2)	C301—P3—C307—C308	−99.9 (2)
C112—C107—C108—C109	−0.3 (5)	Ag2—P3—C307—C308	26.5 (2)
P1—C107—C108—C109	−179.2 (3)	C312—C307—C308—C309	−2.8 (4)
C107—C108—C109—C110	−0.1 (5)	P3—C307—C308—C309	176.3 (2)
C108—C109—C110—C111	−0.3 (5)	C307—C308—C309—C310	1.8 (5)
C109—C110—C111—C112	1.2 (5)	C308—C309—C310—C311	0.2 (5)
C108—C107—C112—C111	1.1 (5)	C309—C310—C311—C312	−1.3 (4)
P1—C107—C112—C111	−179.9 (3)	C308—C307—C312—C311	1.7 (4)
C110—C111—C112—C107	−1.6 (5)	P3—C307—C312—C311	−177.4 (2)
C101—P1—C113—C118	−110.7 (2)	C310—C311—C312—C307	0.3 (4)
C107—P1—C113—C118	−0.8 (2)	C307—P3—C313—C314	113.2 (3)
Ag1—P1—C113—C118	121.3 (2)	C301—P3—C313—C314	4.7 (3)
C101—P1—C113—C114	71.3 (2)	Ag2—P3—C313—C314	−120.7 (3)
C107—P1—C113—C114	−178.8 (2)	C307—P3—C313—C318	−69.9 (3)
Ag1—P1—C113—C114	−56.8 (3)	C301—P3—C313—C318	−178.4 (3)
C118—C113—C114—C115	0.8 (4)	Ag2—P3—C313—C318	56.1 (3)
P1—C113—C114—C115	179.0 (2)	C318—C313—C314—C315	1.2 (5)
C113—C114—C115—C116	−0.5 (5)	P3—C313—C314—C315	178.2 (3)
C114—C115—C116—C117	−0.3 (5)	C313—C314—C315—C316	−0.2 (5)
C115—C116—C117—C118	0.6 (4)	C314—C315—C316—C317	1.1 (5)
C114—C113—C118—C117	−0.5 (4)	C315—C316—C317—C318	−3.1 (5)
P1—C113—C118—C117	−178.6 (2)	C316—C317—C318—C313	4.3 (5)
C116—C117—C118—C113	−0.2 (4)	C314—C313—C318—C317	−3.3 (5)
C207—P2—C201—C206	22.9 (3)	P3—C313—C318—C317	179.6 (3)
C213—P2—C201—C206	−86.1 (3)	C14—O11—C11—C12	6.2 (5)
Ag1—P2—C201—C206	148.6 (2)	O11—C11—C12—C13	−8.9 (6)
C207—P2—C201—C202	−157.5 (2)	C11—C12—C13—C14	8.2 (6)
C213—P2—C201—C202	93.5 (3)	C11—O11—C14—C13	−1.3 (5)
Ag1—P2—C201—C202	−31.8 (3)	C12—C13—C14—O11	−4.5 (6)
C206—C201—C202—C203	2.5 (5)		
